# GINS4 might be a novel prognostic immune-related biomarker of not only esophageal squamous cell carcinoma and other cancers

**DOI:** 10.1186/s12920-022-01223-x

**Published:** 2022-04-01

**Authors:** Donghui Jin, Ligong Yuan, Feng Li, Shuaibo Wang, Yousheng Mao

**Affiliations:** Department of Thoracic Surgery, National Cancer Center/National Clinical Research Center for Cancer/Cancer Hospital, Chinese Academy of Medical Sciences and Peking Union Medical College, Beijing, 100021 China

**Keywords:** Gene Expression Omnibus (GEO), The Cancer Genome Atlas (TCGA), Prognosis, Immune-cell infiltration, Biomarker, GINS4, Pan-cancer

## Abstract

**Background:**

Immunotherapy using immune checkpoint inhibitors (ICIs), such as antibody of programmed death-1 (PD-1)/programmed death-ligand 1 (PD-L1) has showed as a promising treatment for esophageal squamous cell carcinoma (ESCC), but resistance is unavoidable. This study aimed to find more immune-related genes to promote the efficiency of immunotherapy.

**Materials and methods:**

Three datasets were downloaded from Gene Expression Omnibus (GEO) database. Gene differential analysis was performed to identify differentially expressed genes (DEGs), then ceRNA network was constructed based on differentially expressed lncRNAs and mRNAs. Next, Functional enrichment analysis and protein–protein interaction (PPI) network were built to reveal the potential function of mRNAs in ceRNA network. Survival analysis and immune cell infiltration level analysis were utilized to identify prognostic immune-related genes. Finally, pan-cancer analysis was performed to show the role of immune-related genes in other cancers.

**Results:**

The data of 215 samples in total were obtained from GEO database (98 normal tissues and 117 tumor tissues), and 1685 differentially expressed mRNAs (176 downregulated and 1509 upregulated) and 3 upregulated lncRNAs (MCM3AP-AS1, HCP5 and GUSBP11, all upregulated) were found. ceRNA network was constructed to reveal some special correlation. Function enrichment showed some potential functions of mRNAs in ceRNA network such as mitotic cell cycle process, negative regulation of DNA-binding transcription factor activity, ossification, VEGFA-VEGFR2 signaling pathway, epithelial to mesenchymal transition, embryonic morphogenesis and so on. PPI network showed the physical interactions between each mRNA in ceRNA network. Through survival analysis and immune cell infiltration level analysis, GINS4 was confirmed as an immune-related prognostic gene in ESCC. GSEA showed some potential functions such as negative regulation of monocyte chemotaxis, antigen processing and presentation of endogenous peptide antigen via MHC class I via ER pathway, positive regulation of antigen processing and presentation, dendritic cell antigen processing and presentation and so on. Finally, pan-cancer analysis revealed that GINS4 might be a novel immune-related prognostic gene in ESCC and other cancers.

**Conclusion:**

Our study suggested that GINS4 was correlated with prognosis and immune cell infiltration level of ESCC and other cancers. It may deserve further investigation as a potential immune-related prognostic biomarker of ESCC and other cancers.

## Introduction

Esophageal cancer (EC) is one of the most common malignant tumors with a high incidence and mortality in the world [1]. It consists of two major histological types, esophageal squamous cell carcinoma (ESCC) and esophageal adenocarcinoma (EAC). ESCC is more commonly seen than EAC in the eastern countries [2]. Surgery or surgery-based multimodality therapy is currently the most effective treatment for ESCC [3]. For locally advanced ESCC, neoadjuvant chemotherapy combined with radiotherapy or immunotherapy is now the mainstay for improvement of survival [4–6].

Though great progress has been made in the biological functions of driver gene for esophageal cancer, there are still lack of effective targeted drugs for ESCC. In recent years, immune checkpoint inhibitors (ICIs), such as the antibody of programmed death-1 (PD-1)/programmed death-ligand 1 (PD-L1) has showed its promising efficiency as neoadjuvant or adjuvant therapy, especially when combined with chemotherapy. Studies have shown that PD-1/PD-L1 inhibitors such as nivolumab, pembrolizumab can significantly improve the objective response ratio (ORR) with a good safety and prolong over survival (OS) in both first-line and second-line treatment for advanced ESCC [7–10]. In fact, according to some phase I-III trials, though ICIs have showed its effectiveness, only a subset of patients would benefit from them [11]. For example, the overall response rate (ORR) was 30% in in KEYNOTE-028 trial [9] and 9.9% in KEYNOTE-180 trial [12]. What’s more, ATTRACTION-3 trial, a multicenter, randomized phase III trial, showed that the ORR was only 19% [13]. Therefore, it’s urgent to explore more immunotherapy approaches to benefit more patients.

Studies have shown that tumor immune microenvironment (TIME) including tumor‐infiltrating lymphocytes (TIL), tumor‐associated macrophages (TAM), and myeloid‐derived suppressor cells (MDSC) may contribute to the resistance to immunotherapy, but the mechanisms are still unknown [14–17]. Therefore, immune-related genes and immunotherapy-related biomarkers are urgently needed, which may help to elucidate the molecular mechanisms of TIME on tumorigenesis, and also improve the efficiency of immunotherapy. In this study, we aimed to search for novel immune-related genes through integrated analysis on the data downloaded from Gene Expression Omnibus (GEO) database and The Cancer Genome Atlas (TCGA) database.

## Materials and methods

### Gene sets acquisition

The data of ESCC datasets GSE33426 (normal: 12, tumor: 59), GSE38129 (normal: 30, tumor: 30) and GSE161533 (normal: 56, tumor: 28) were downloaded from GEO database. Then the three datasets were merged into one new dataset, and package “sva” in software R (ver. 4.1.0) was utilized to remove the batch effect. Next, the merged dataset was divided the into mRNA group and lncRNA group according to the GENCODE project (http://www.gencodegenes.org).

### Gene differential analysis

In order to find out the differentially expressed genes (DEGs), the gene differential analysis was performed between tumor and normal tissues on mRNA group and lncRNA group by “limma” package. |LogFC|> 1 and adjusted *p* value < 0.05 were considered statistically significant for the DEGs.

### Construction of ceRNA network

In order to find out the competing endogenous regulating network mediated by lncRNAs and miRNAs, we constructed a ceRNA network by software Cytoscape (ver. 3.8.2). First, we predicted the potential miRNAs which could correlate with differentially expressed lncRNAs by miRcode database (http://www.mircode.org/) [18]. Next, the interactions between miRNAs and differentially expressed mRNAs were predicted by miRTarBase (http://miRTarBase.cuhk.edu.cn/) [19], TargetScan (http://www.targetscan.org/) [20] and miRDB (http://www.mirdb.org/miRDB/) [21]. The interactions between miRNAs and mRNAs should match all the three databases. Software Cytoscape (ver. 3.8.2) was used to visualize the network.

### Functional enrichment analysis and protein–protein interaction network

In order to reveal the potential function of mRNA in ceRNA network, we uploaded the mRNAs in ceRNA network to the website Metascape (https://metascape.org/gp) [22]. In this website, we could perform function enrichment analysis including gene ontology (GO) analysis, Kyoto Encyclopedia of Genes and Genomes (KEGG) pathway analysis and protein–protein interaction (PPI) enrichment analysis.

### Survival analyses

Due to lack of clinicopathological features in GEO datasets, we downloaded ESCC gene expression profile and corresponding clinicopathological features from The Cancer Genome Atlas (TCGA) (https://portal.gdc.cancer.gov/). Then survival analyses were carried out based on the lncRNAs and mRNAs in ceRNA network. Kaplan–Meier (KM) survival curves were constructed by “survival” and “survminer” packages. Log-rank *p* < 0.05 indicated a significance difference.

### Immune cell infiltration level

To explore whether the prognostic mRNAs were correlated with TIME, we uploaded tumor expression profile of GEO dataset to Tumor IMmune Estimation Resource (TIMER) database (https://cistrome.shinyapps.io/timer/) to calculate six immune cell infiltration level (B cell, T cell CD4+, T cell CD8+, Neutrophil, Macrophage and Myeloid dendritic cell) [23]. Spearman analyses were performed to reveal the correlation between the expression level of prognostic mRNAs and six immune cell infiltration level. A significance difference was indicated when *p* < 0.05 and the mRNAs were confirmed as immune-related mRNAs. Furthermore, we also performed Estimation of STromal and Immune cells in MAlignant Tumor tissues using Expression data (ESTIMATE) analysis based on tumor expression profile of GEO dataset by using “estimate” package to calculate immune score, then Spearman analyses were performed between immune score and immune-related genes to validate the correlation between immune score and immune-related mRNAs. A significance difference was indicated when *p* < 0.05. Correlations between expression level of immune-related mRNAs and biomarkers of immune cells were also revealed. The biomarkers were obtained from previous study [24] and CellMarker database (http://biocc.hrbmu.edu.cn/CellMarker/index.jsp) [25].

### Gene set enrichment analysis

To reveal the potential function of immune-related genes in ESCC tumor, we carried out Gene set enrichment analysis (GSEA) based on each immune-related mRNA by software GSEA (ver. 4.1.0). The tumor expression profile was divided into high expression groups and low expression group based on the median expression level of the aimed mRNA each time when a GSEA was performed. GSEA was conducted based on the high and low express groups. A significant functional enrichment was indicated if *p* < 0.05.

### Pan-cancer analysis

In order to search out the role of the immune-related prognostic genes found in the ESCC in other cancers, a pan-cancer analysis was conducted, including gene differential analysis, survival analysis and immune cell infiltration analysis. Gene differential analysis and immune cell infiltration analysis were performed via TIMER database, and survival analysis was carried out by GEPIA database (http://gepia.cancer-pku.cn/index.html).

## Results

### Gene expression profile data

**Fig. 1:**
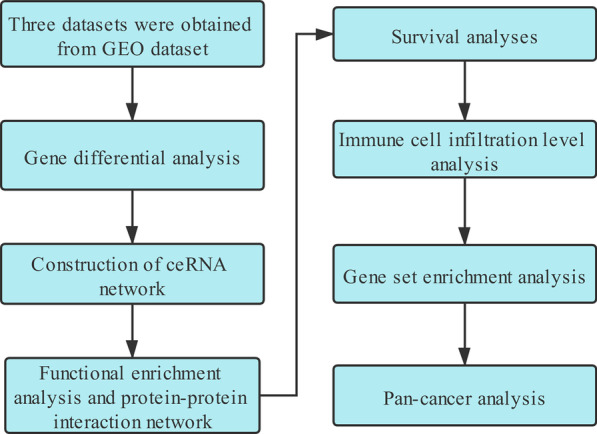
Flowchart of our study

**Fig. 2:**
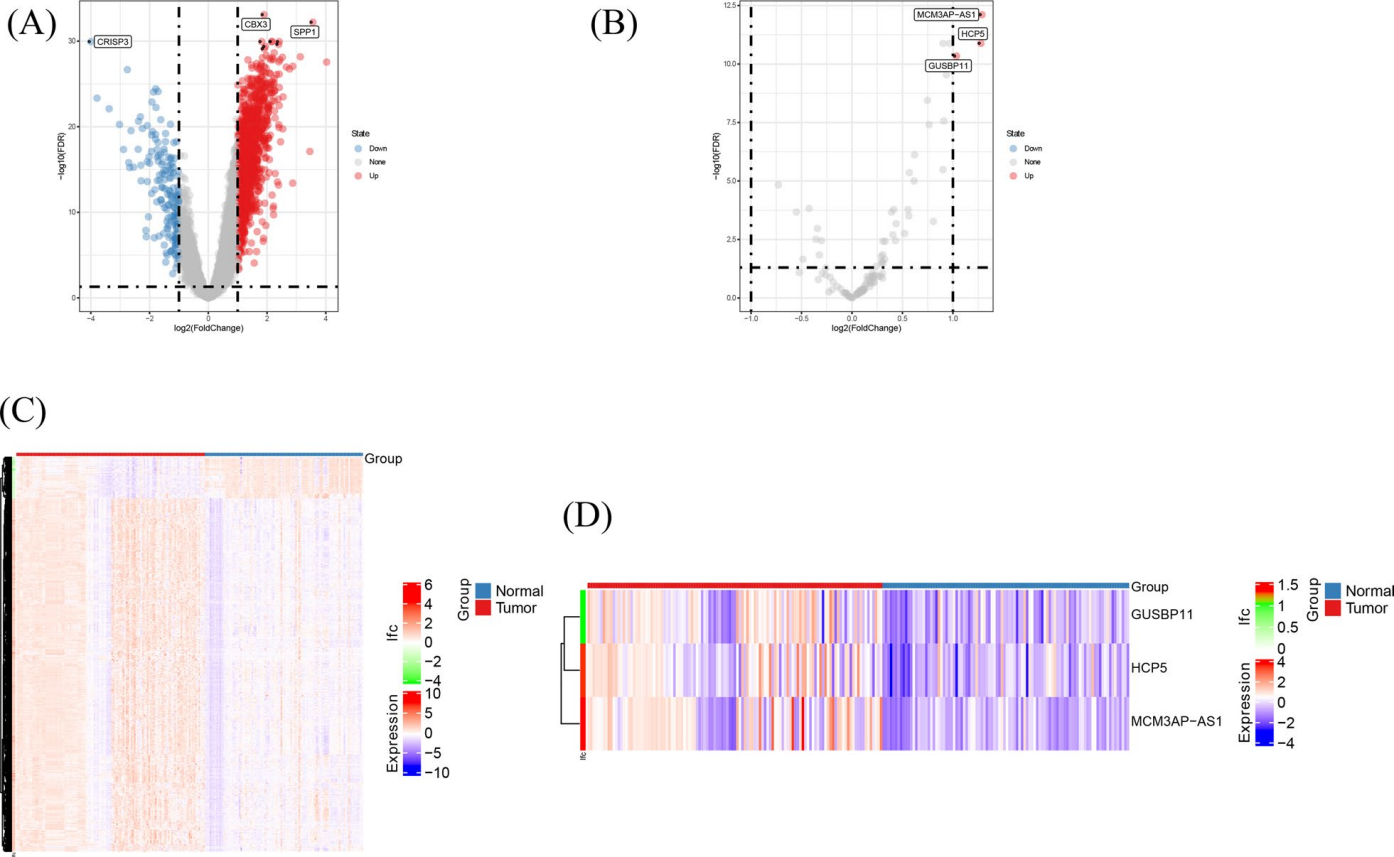
Volcano plots and heatmap plots of differential expressed mRNAs (**A**, **C**) and lncRNAs (**B**, **D**)

The work flow of this study was shown in Fig. 1. The data of 215 samples in total were obtained from GEO database (98 normal tissues and 117 tumor tissues). After normalization, gene differential analysis was conducted. According to |LogFC|> 1 and adjusted *p* value < 0.05, in mRNA group, 1685 mRNAs were differentially expressed (176 were downregulated and 1509 were upregulated); in lncRNA group, only 3 lncRNAs (MCM3AP-AS1, HCP5 and GUSBP11) were differentially expressed and all upregulated (Fig. 2A–D). The results of gene differential analysis were showed by volcano plots and the gene expression was showed by heatmaps.

### ceRNA network

**Fig. 3:**
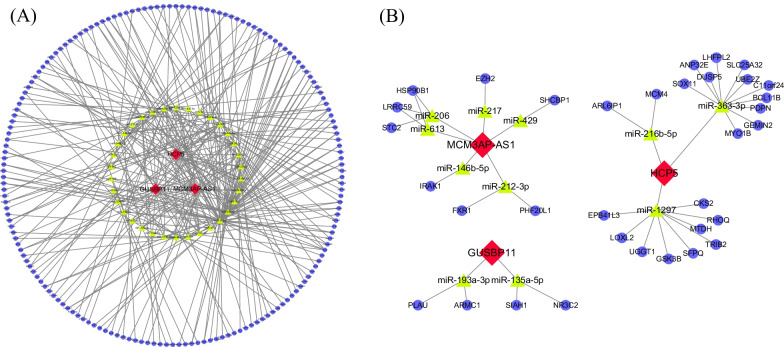
ceRNA network constructed based on differential expressed lncRNAs and mRNAs. **A** The whole ceRNA network. **B** Three special ceRNA networks

After prediction, 3 lncRNAs, 34 miRNAs and 169 mRNAs were included in the ceRNA network (Fig. 3A). In the ceRNA network, lncRNAs could regulate downstream mRNAs by regulating correlated miRNAs [26]. Three special networks were showed in the Fig. 3B. In these three networks, miRNAs only correlated with one lncRNA, and mRNAs only correlated with one miRNA. These networks were unique, and showed special functions in ESCC.

### Functional enrichment analysis and PPI network

**Fig. 4:**
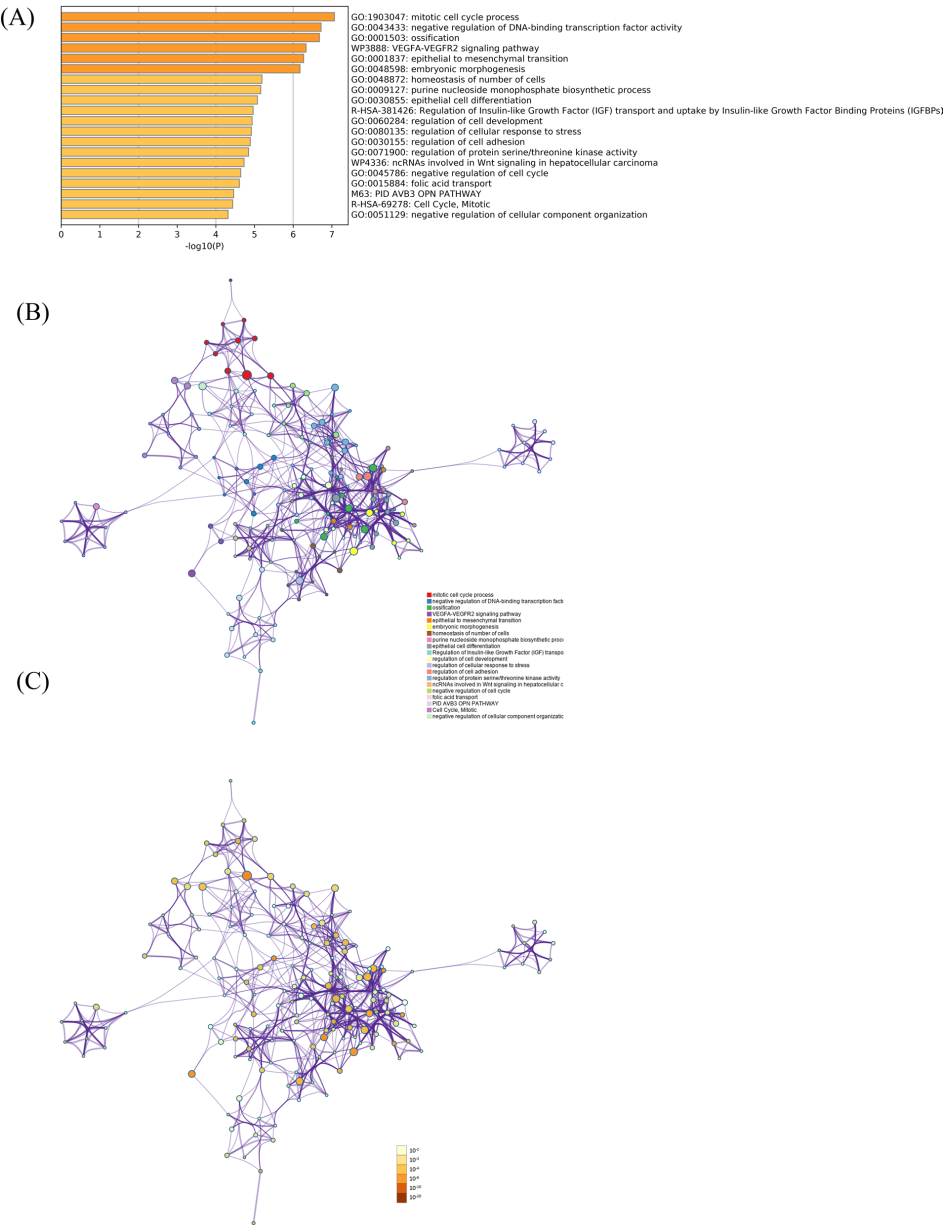
Results of function enrichment analysis. **A** Potential function of mRNAs in ceRNA network. **B**–**C** Network of enriched terms: **B** colored by cluster ID, where nodes that share the same cluster ID are typically close to each other; **C** colored by p-value, where terms containing more genes tend to have a more significant *p* value

**Fig. 5:**
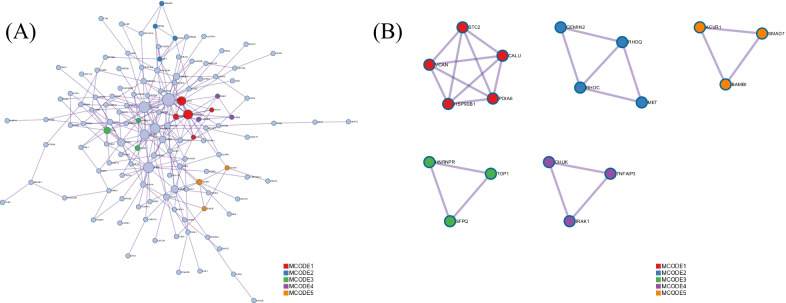
**A** Protein–protein interaction (PPI) network. **B** The Molecular Complex Detection (MCODE) components. Pathway and process enrichment analysis has been applied to each MCODE component independently, and the three best-scoring terms by p-value have been retained as the functional description of the corresponding components

Functional enrich analysis showed that mRNAs in ceRNA network were mainly enriched in mitotic cell cycle process, negative regulation of DNA-binding transcription factor activity, ossification, VEGFA-VEGFR2 signaling pathway, epithelial to mesenchymal transition, embryonic morphogenesis and so on (Fig. 4A). Some functions such as VEGFA-VEGFR2 signaling pathway, epithelial to mesenchymal transition, epithelial cell differentiation, regulation of cell development, regulation of cell adhesion may contribute to the tumorigenesis and metastasis of ESCC. Furthermore, enriched functions with a similarity > 0.3 were connected by edges (Fig. 4B–C). PPI network showed the physical interactions between each mRNA in ceRNA network. The Molecular Complex Detection (MCODE) algorithm has been applied to identify densely connected network components (Fig. 5A–B). Pathway and process enrichment analysis has been applied to each MCODE component independently, and the three best-scoring terms by p-value have been retained as the functional description of the corresponding components (Table 1).

**Table 1:** The three best-scoring terms by p-value retained as the functional description of the corresponding components in PPI network

MCODE	GO	Description	Log10(P)
MCODE_1	R-HSA-8957275	Post-translational protein phosphorylation	− 12.1
MCODE_1	R-HSA-381426	Regulation of Insulin-like Growth Factor (IGF) transport and uptake by Insulin-like Growth Factor Binding Proteins (IGFBPs)	− 11.8
MCODE_2	GO:0032956	regulation of actin cytoskeleton organization	− 5.1
MCODE_2	GO:0032970	regulation of actin filament-based process	− 5
MCODE_2	GO:0007264	small GTPase mediated signal transduction	− 4.7
MCODE_3	GO:0007623	circadian rhythm	− 6.4
MCODE_3	GO:0048511	rhythmic process	− 5.9
MCODE_4	R-HSA-168638	NOD1/2 Signaling Pathway	− 8.7
MCODE_4	R-HSA-168643	Nucleotide-binding domain, leucine rich repeat containing receptor (NLR) signaling pathways	− 8.1
MCODE_4	GO:0032088	negative regulation of NF-kappaB transcription factor activity	− 7.5
MCODE_5	hsa04350	TGF-beta signaling pathway	− 7.6
MCODE_5	ko04350	TGF-beta signaling pathway	− 7.6
MCODE_5	GO:0010717	regulation of epithelial to mesenchymal transition	− 7.4

### KM survival curves from TCGA database

**Fig. 6:**
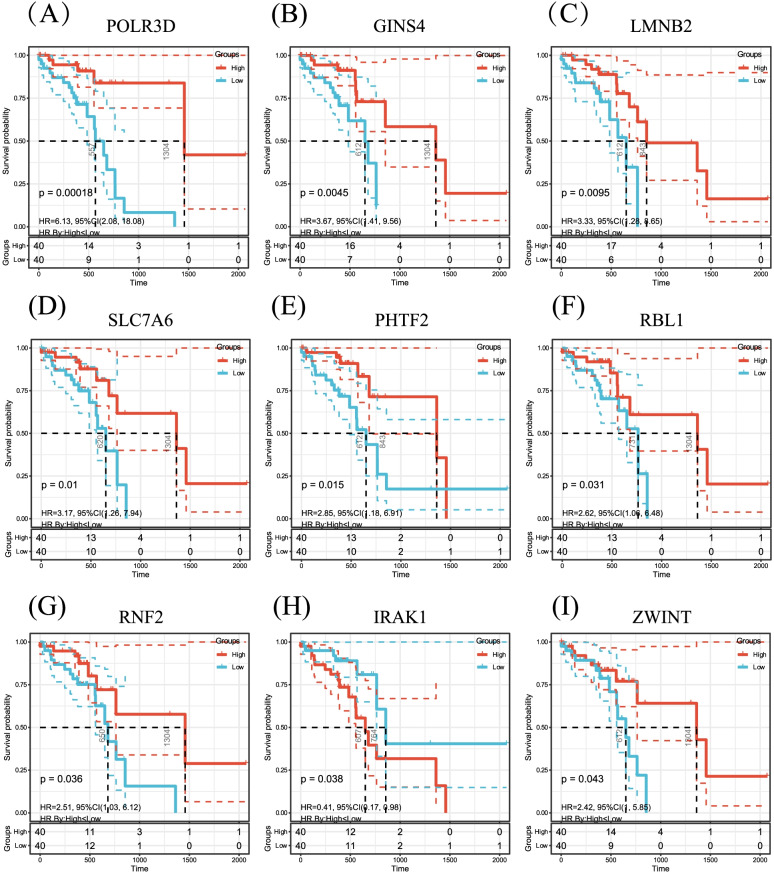
Kaplan–Meier (KM) curves of the mRNAs in ceRNA network were built based on The Cancer Genome Atlas (TCGA) database and 9 mRNAs had prognostic value (**A**–**I**)

The data of 80 patients’ tumor samples with clinicopathological features were obtained from TCGA database, of those 56 were still alive and 24 dead with a median survival time of 395.5 days. Survival analyses was performed and KM curves was built on the 3 lncRNAs and 169 mRNAs in ceRNA network. The results showed that all 3 lncRNAs were not prognostic while 9 upregulated mRNAs (POLR3D, GINS4, LMNB2, SLC7A6, PHTF2, RBL1, RNF2, IRAK1 and ZWINT) were prognostic (Fig. 6A–I).

### Correlation between immune cell infiltration level and prognostic mRNAs

**Fig. 7:**
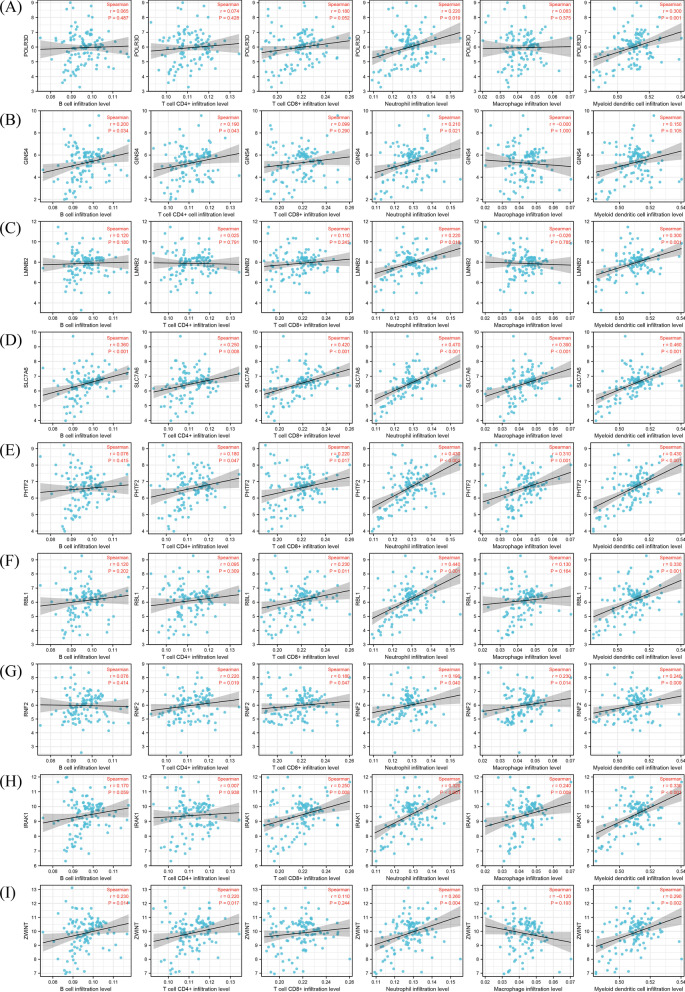
Correlation between the 9 mRNAs and immune cell infiltration level based on GEO dataset. All the 9 mRNAs were correlated with the infiltration level of some immune cells. (**A**-**I**: POLR3D, GINS4, LMNB2, SLC7A6, PHTF2, RBL1, RNF2, IRAK1 and ZWINT)

**Fig. 8:**
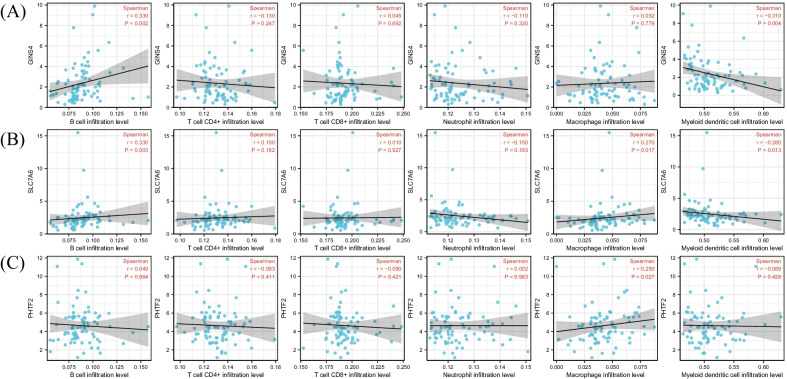
Correlation between the 9 mRNAs and immune cell infiltration level based on TCGA dataset. Results showed only GINS4, SCL7A6 and PHTF2 were correlated with the infiltration level of some immune cells. **A** GINS4; **B** SCL7A6; **C** PHTF2

Six immune cell infiltration level were acquired from TIMER database. Correlation analyses between six immune cell infiltration level and prognostic mRNAs were conducted. Results showed that all 9 mRNAs were correlated with somewhat infiltration level of the 6 immune cells (Fig. 7A–I). To validate these results, we also uploaded tumor expression profile of TCGA dataset to TIMER database, and the same analyses were performed. Results showed that only GINS4, PHTF2 and SLC7A6 were correlated with immune cell infiltration. GINS4 was correlated with the infiltration of B cell and myeloid dendritic cell; PHTF2 was correlated with the infiltration of macrophage; SLC7A6 was correlated with the infiltration of B cell, macrophage and myeloid dendritic cell (Fig. 8A–C). Both datasets showed that GINS4 was correlated with B cell infiltration, and GEO dataset showed GINS4 was also correlated with the infiltration of neutrophil and T cell CD4+; TCGA dataset showed GINS4 was also correlated with the infiltration of myeloid dendritic cell. In GEO dataset, PHTF2 was correlated with the infiltration of all immune cells except B cell; however, in TCGA dataset, PHTF2 was only correlated with macrophage. In GEO dataset, SLC7A6 was correlated with the infiltration of all immune cells, but in TCGA dataset, it only correlated with infiltration of 3 immune cells. In conclusion, the above results indicated that GINS4, SLC7A6 and PHTF2 might be immune-related genes. ESTIMATE analysis was conducted for validating the results, and correlation analyses between immune-related genes and immune score were performed. The results showed that only GINS4 was correlated with immune score (r = − 0.3, p = 0.001), while SLC7A6 (r = 0.170, p = 0.069) and PHTF2 (r = − 0.020, p = 0.831) were excluded. Therefore, GINS4 was confirmed as an immune-related gene.

### Correlation of GINS4 expression with biomarkers of immune cells

To further explore the role of GINS4 in tumor immune, we determined the expression correlation of GINS4 with biomarkers of B cell, CD4 + T cell, neutrophil, dendritic cell and myeloid dendritic cell based on GEO datasets. As list in Table 2, GINS4 was significantly correlated with B cell’s biomarker (CD19 and CD79A), one biomarker of dendritic cell (HLA-DQB1) and 5 biomarkers of myeloid dendritic cell (CD40, CD80, CD83, CD207 and CD209). These findings partially supported that GINS4 is positively correlated with immune cell infiltration.

**Table 2:** Correlation between GINS4 and biomarker of immune cells

Immune cell	Biomarker	R	*P*
B cell	CD19	0.2	**0.029**
	CD79A	0.2	**0.034**
Neutrophil	CEACAM8	0.16	0.081
	ITGAM	0.18	0.056
	CCR7	0.12	0.192
CD4+ T cell	CD4	0.13	0.177
Dendritic cell	HLA-DPB1	0.086	0.356
	HLA-DQB1	0.28	**0.003**
	HLA-DRA	0.14	0.14
	HLA-DPA1	0.055	0.558
	CD1C	0.15	0.098
	NRP1	− 0.073	0.436
	ITGAX	0.14	0.13
Myeloid dendritic cell	CD4	0.13	0.177
	CD40	0.36	** < 0.001**
	CD80	0.38	** < 0.001**
	CD83	0.37	** < 0.001**
	CD86	0.064	0.49
	CD207	0.36	** < 0.001**
	CD209	0.24	**0.009**

The bold means *p* < 0.05

### Potential function of GINS4

**Fig. 9:**
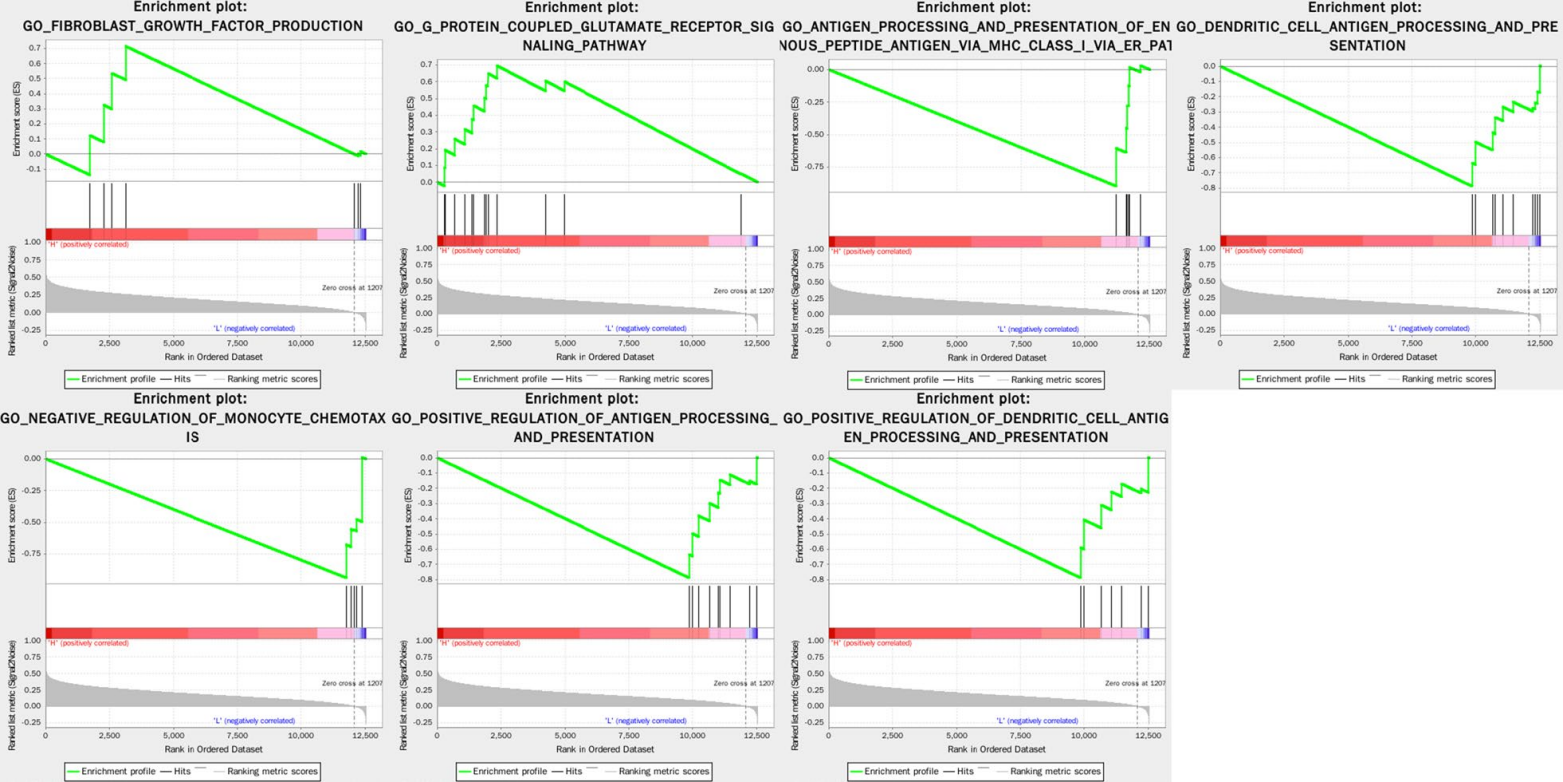
Gene set enrichment analysis (GSEA) showed some potential function of GINS4. These functions might correlate with the tumorigenesis and immunity of esophageal squamous cell carcinoma (ESCC)

The expression profiles of the tumors were divided into high expression and low expression group based on the median expression level of GINS4, and GSEA was carried out based on the two groups. The results showed that high expression group was mainly enriched in G Protein coupled glutamate receptor signaling pathway and fibroblast growth factor production; Low expression group was mainly enriched in negative regulation of monocyte chemotaxis, antigen processing and presentation of endogenous peptide antigen via MHC class I via ER pathway, positive regulation of antigen processing and presentation, dendritic cell antigen processing and presentation, and positive regulation of dendritic cell antigen processing and presentation (Fig. 9). These results demonstrated the role of GINS4 in ESCC tumor immune.

### Pan-cancer analysis of GINS4

**Fig. 10:**
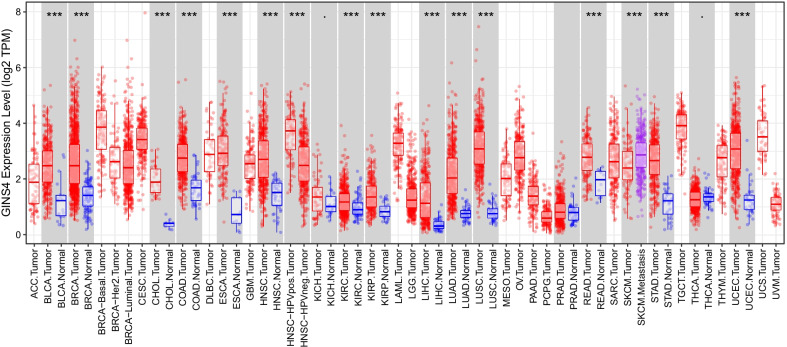
The expression level of GINS4 in different cancers and adjacent normal tissues analyzed by Tumor IMmune Estimation Resource (TIMER) database. *p < 0.05; **p < 0.01; ***p < 0.001

**Fig. 11:**
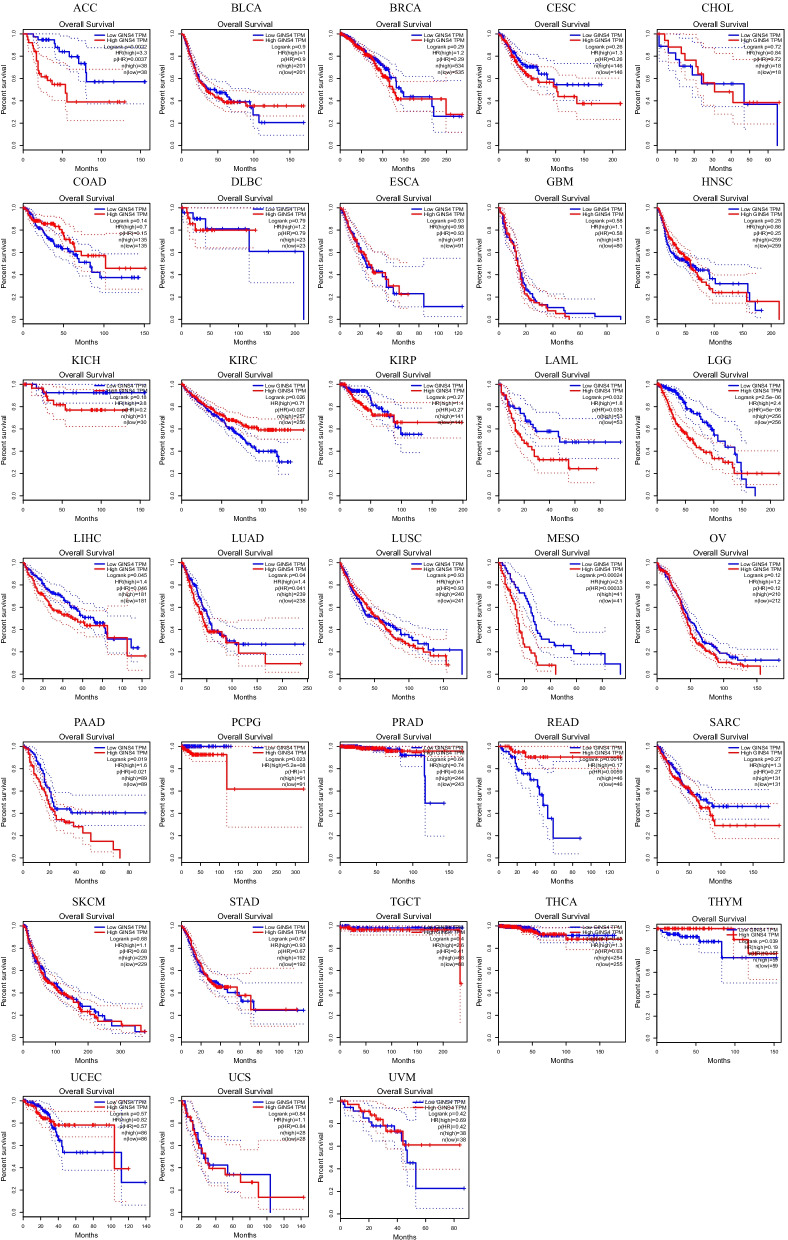
Kaplan–Meier curves of GINS4 in different cancers from GEPIA database. Results showed that GINS4 had prognostic value in adrenocortical carcinoma (ACC, *p* = 0.0022), kidney renal clear cell carcinoma (KIRC, *p* = 0.026), acute myeloid leukemia (LAML, *p* = 0.032), brain lower grade glioma (LGG, *p* = 2.5e−06), liver hepatocellular carcinoma (LIHC, *p* = 0.045), lung adenocarcinoma (LUAD, *p* = 0.04), mesothelioma (MESO, *p* = 0.00024), pancreatic adenocarcinoma (PAAD, *p* = 0.019), pheochromocytoma and paraganglioma (PCPG, *p* = 0.023), READ (*p* = 0.0019) and thymoma (THYM, *p* = 0.039)

**Fig. 12:**
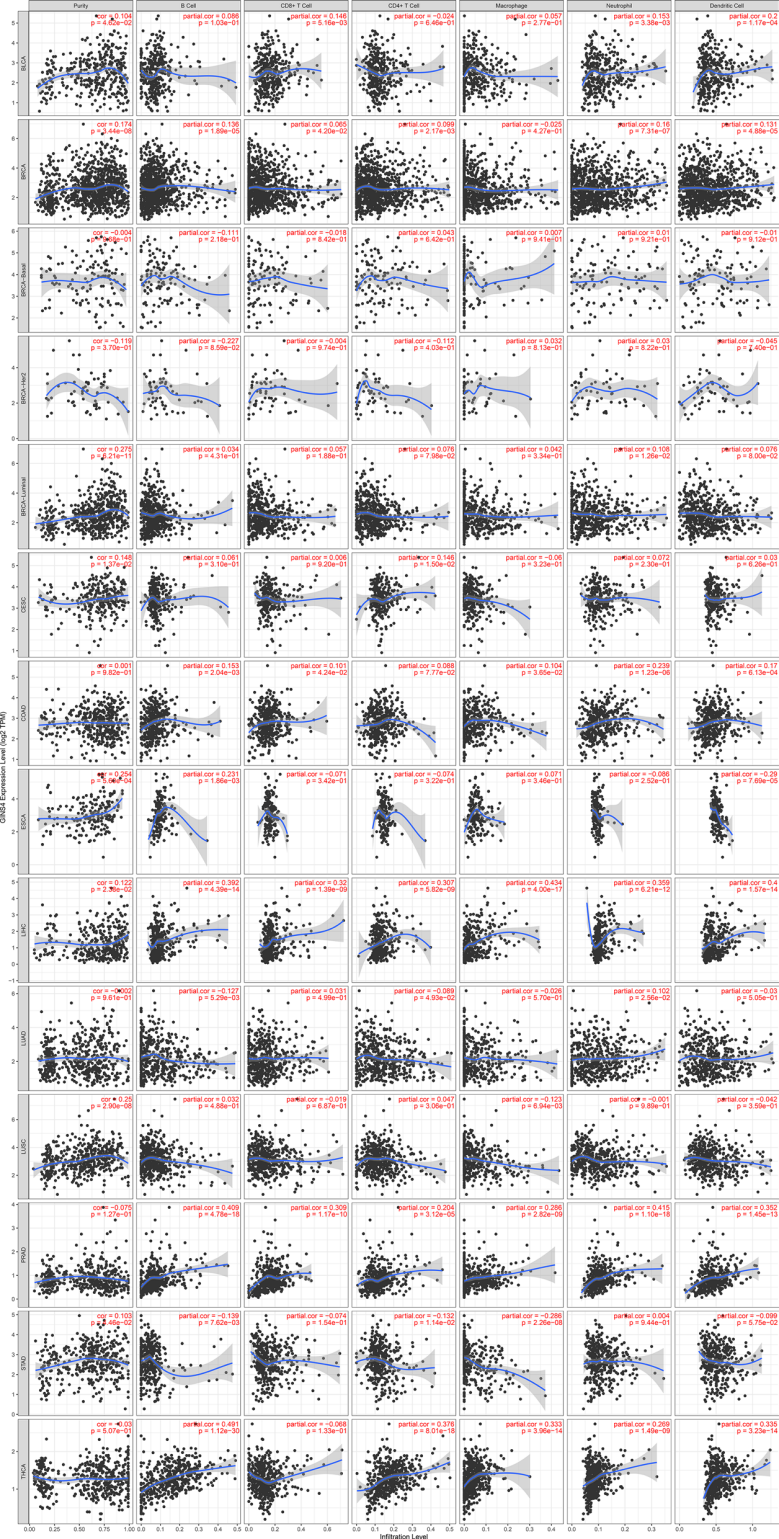
Immune cell infiltration analysis was based on the top 10 cancer in global morbidity via TIMER database. GINS4 was correlated with immune cell infiltration in all cancers, especially in bladder urothelial carcinoma (BLCA), breast invasive carcinoma (BRCA), colon adenocarcinoma (COAD), liver hepatocellular carcinoma (LIHC), lung adenocarcinoma (LUAD), prostate adenocarcinoma (PRAD), stomach adenocarcinoma (STAD), thyroid carcinoma (THCA)

Gene differential analysis was performed in 33 types of cancer by TIMER database. The results showed that GINS4 was differentially expressed in bladder urothelial carcinoma (BLCA), breast invasive carcinoma (BRCA), cholangiocarcinoma (CHOL), colon adenocarcinoma (COAD), esophageal carcinoma (ESCA), head and neck squamous cell carcinoma (HNSC, including HNSC-HPV positive and negative), kidney renal clear cell carcinoma (KIRC), kidney renal papillary cell carcinoma (KIRP), liver hepatocellular carcinoma (LIHC), lung adenocarcinoma (LUAD), Lung squamous cell carcinoma (LUSC), rectum adenocarcinoma (READ), skin cutaneous melanoma (SKCM), stomach adenocarcinoma (STAD) and uterine corpus endometrial carcinoma (UCEC) (Fig. 10). KM curves for 33 cancers were obtained from GEPIA database. The results showed that GINS4 had prognostic value in adrenocortical carcinoma (ACC, p = 0.0022), KIRC (p = 0.026), acute myeloid leukemia (LAML, p = 0.032), brain lower grade glioma (LGG, p = 2.5e−06), LIHC (p = 0.045), LUAD (p = 0.04), mesothelioma (MESO, p = 0.00024), pancreatic adenocarcinoma (PAAD, p = 0.019), pheochromocytoma and paraganglioma (PCPG, p = 0.023), READ (p = 0.0019) and thymoma (THYM, p = 0.039) (Fig. 11). Immune cell infiltration analysis was based on the top 10 cancer in global morbidity via TIMER database. GINS4 was correlated with immune cell infiltration in all cancers, especially in BLCA, BRCA, COAD, LIHC, LUAD, prostate adenocarcinoma (PRAD), STAD, thyroid carcinoma (THCA) (Fig. 12). In conclusion, GINS4 might be a novel immune-related prognostic gene in ESCC and other cancers.

## Discussion

In recent years, immunotherapy for esophageal cancer is developing rapidly, and has showed very promising results in the neoadjuvant and or adjuvant therapy for ESCC. Therefore, in order to predict the efficiency and prognosis of the esophageal cancer patients receiving immunotherapy, a lot of studies has been conducted on searching for immune-related genes. In this study, we systematically collected data from GEO database, and performed gene differential analysis. ceRNA network was constructed based on DEGs, and function enrichment and PPI network were performed based on DEGs in ceRNA network. Then, survival analysis and immune cell infiltration level analysis were carried out to search for immune-related prognostic genes. The results showed that GINS4 was immune-related prognostic gene and GSEA revealed its potential function. We also performed a pan-cancer analysis to investigate the role of GINS4 in other cancers.

ceRNA networks link the function of protein-coding mRNAs with that of non-coding RNAs such as microRNA, long non-coding RNA, pseudogenic RNA and circular RNA. lncRNAs could competitively bind to the shared miRNAs, and their expression is then positively correlated. The upregulation of one lncRNA results in more sequestrated copies of shared miRNAs [26]. In our study, ceRNA network was constructed by DEGs, indicating that the regulation network plays a role in ESCC, especially the 3 unique networks in Fig. 2B.

PPI enrichment analysis revealed the physical interactions among DEGs in ceRNA network, and MCODE showed the potential protein complex and their functions. Some functions such as negative regulation of NF-kappaB transcription factor activity [27], TGF-beta signaling pathway [28] may contribute to tumorigenesis of ESCC.

Our study found that GINS4 might be a novel biomarker correlated with the prognosis not only in ESCC but also in many other cancers. Go, Ichi, Nii, and San (means five, one, two, and three, respectively, in Japanese) complex subunit 4 (GINS4) is a member of GINS family, which are essential for the initiation of DNA replication in yeast and Xenopus egg extracts [29]. Yang et al. reported that GINS4 was highly expressed in non-small cell lung cancer (NSCLC) and was associated with the prognosis of NSCLC, especially LUAD. They also found that overexpression of GINS4 promotes cancer cell growth, migration and invasion [30]. Zhu et al. found that GINS4 was highly expressed in gastric cancer and correlated closely with gastric cancer clinicopathological features such as OS and disease-free survival (DFS) [31]. Similar results were also found in colorectal cancer [32], pancreatic cancer [33] and hepatocellular carcinoma [34] and breast cancer [35].

In ceRNA network, GINS4 was regulated via HCP5/miR-17-5p axis. Histocompatibility leukocyte antigen complex P5 (HCP5) is a lncRNA located between the MICA (MHC class I polypeptide-related sequence A) and MICB (MHC class I polypeptide-related sequence B) genes in the MHC I region of chromosome 6p21.33, and it is mainly expressed in immune system. HCP5 was associated with tumorigenesis of many cancers such as pancreatic cancer, colorectal cancer, lung cancer and so on [36]. Thus, HCP5 may function by regulating GINS4.

miR-17-5p is also regulated by other lncRNAs or target other mRNAs and then, promotes tumorigenesis, for example, MIR17HG promotes colorectal cancer progression via miR-17-5p [37], miR-17-5p promotes angiogenesis in nasopharyngeal carcinoma via targeting BAMBI [38]. Up to now, there are no studies about HCP5 / miR-17-5p/GINS4 axis.

Furthermore, our study also found that GINS4 was correlated with immune cell infiltration not only in ESCC but also in many other cancers. In ESCC, GINS4 was significantly associated with the infiltration level of B cell. It was also correlated with CD4+ T cell and myeloid dendritic cell according to GEO and TCGA dataset. GSEA showed GINS4 might have an important role in immune system. Therefore, GINS4 was confirmed as an immune-related prognostic gene in ESCC. However, no related researches have reported it until present.

The limitation of this study is that the GINS4 was confirmed as an immune-related prognostic gene in ESCC based on the analysis of the data downloaded from GEO and TCGA database. Further molecular biology experiments are required to investigate its function and regulation mechanism.

## Conclusion

Our study found that GINS4 might be a novel immune-related prognostic gene in ESCC. It is highly expressed in ESCC, and may be regulated via HCP5 / miR-17-5p axis. It may also play an important role in other cancers. Therefore, it could be a new target gene, which provides a new therapeutic target in many malignant tumors. Further studies are required to investigate its function.

## Data Availability

All the datasets analyzed for this study can be found in the GEO database (https://www.ncbi.nlm.nih.gov/gds/), TCGA database (https://portal.gdc.cancer.gov/), TIMER database (https://cistrome.shinyapps.io/timer/) and GEPIA database (http://gepia.cancer-pku.cn/index.html).

## Abbreviations

EC: Esophageal cancer
ESCC: Esophageal squamous cell carcinoma
EAC: Esophageal adenocarcinoma
ICIs: Immune checkpoint inhibitors
PD-1: Programmed death-1
PD-L1: Programmed death-ligand 1
ORR: Objective response ratio
OS: Over survival
TIME: Tumor immune microenvironment
TIL: Tumor‐infiltrating lymphocytes
TAM: Tumor‐associated macrophages
MDSC: Myeloid‐derived suppressor cells
GEO: Gene Expression Omnibus
TCGA: The Cancer Genome Atlas
DEGs: Differentially expressed genes
GO: Gene ontology
KEGG: Kyoto Encyclopedia of Genes and Genomes
PPI: Protein–protein interaction
KM: Kaplan–Meier
TIMER: Tumor IMmune Estimation Resource
ESTIMATE: Estimation of STromal and Immune cells in MAlignant Tumor tissues using Expression data
GSEA: Gene set enrichment analysis
MCODE: Molecular Complex Detection
BLCA: Bladder urothelial carcinoma
BRCA: Breast invasive carcinoma
CHOL: Cholangiocarcinoma
COAD: Colon adenocarcinoma
ESCA: Esophageal carcinoma
HNSC: Head and neck squamous cell carcinoma
KIRC: Kidney renal clear cell carcinoma
KIRP: Kidney renal papillary cell carcinoma
LIHC: Liver hepatocellular carcinoma
LUAD: Lung adenocarcinoma
LUSC: Lung squamous cell carcinoma
READ: Rectum adenocarcinoma
SKCM: Skin cutaneous melanoma
STAD: Stomach adenocarcinoma
UCEC: Uterine corpus endometrial carcinoma
ACC: Adrenocortical carcinoma
LAML: Acute myeloid leukemia
LGG: Brain lower grade glioma
PAAD: Pancreatic adenocarcinoma
THYM: Thymoma
PRAD: Prostate adenocarcinoma
THCA: Thyroid carcinoma
GINS4: Go, Ichi, Nii, and San complex subunit 4
NSCLC: Non-small cell lung cancer
DFS: Disease-free survival
HCP5: Histocompatibility leukocyte antigen complex P5
MICA: MHC class I polypeptide-related sequence A
MICB: MHC class I polypeptide-related sequence B
